# NDUFAB1 as a Novel Regulator of NEFA-Induced Metabolic Dysfunction in Bovine Adipocytes

**DOI:** 10.3390/ani15111618

**Published:** 2025-05-30

**Authors:** Jing Zhou, Tao Tang, Wenqiang Sun, Xianbo Jia, Jie Wang, Hengwei Yu, Songjia Lai

**Affiliations:** College of Animal Science and Technology, Sichuan Agricultural University, Chengdu 611130, China; 13669919401@163.com (J.Z.); m18483220592@163.com (T.T.); wqsun2021@163.com (W.S.); jaxb369@sicau.edu.cn (X.J.); wjie68@163.com (J.W.); 18792427097@163.com (H.Y.)

**Keywords:** NEFA, bovine, adipocyte, NDUFAB1, metabolic dysfunction

## Abstract

Elevated non-esterified fatty acid (NEFA) levels are closely associated with metabolic disorders in dairy cattle, yet their direct effects on adipocyte physiology remain poorly understood. In this study, we demonstrate that high NEFA concentrations significantly impair bovine adipocyte function by simultaneously inhibiting proliferation/differentiation (*p* < 0.01) and promoting pathological lipid deposition. Through integrated transcriptomic and functional analyses, we identified NDUFAB1 as a central metabolic regulator that counteracts NEFA-induced adipocyte dysfunction. Mechanistically, NDUFAB1 activation attenuates the cytotoxic effects of excessive NEFA exposure. These findings provide both fundamental insights into energy metabolism regulation and a potential therapeutic target (NDUFAB1) for preventing bovine metabolic diseases.

## Simple Summary

Dairy cows often develop health problems when their blood contains too much of a specific type of fat called non-esterified fatty acids (NEFAs), but we do not fully understand how this affects their fat cells (adipocytes). Our study shows that high NEFA levels harm these adipocytes by stopping their normal growth and causing unhealthy fat buildup. We discovered that boosting a key metabolic protein (NDUFAB1) helps adipocytes resist this damage. These findings explain how fat metabolism goes wrong in cows, and suggest a new way to prevent common dairy cattle diseases by targeting NDUFAB1. This research could lead to refine strategies for keeping cows healthier, improving milk production, and reducing costs for farmers.

## 1. Introduction

Non-esterified fatty acids (NEFA), crucial products of energy metabolism, play a pivotal role in the metabolic regulation of cattle, particularly during the periparturient period. This phase is often characterized by a state of negative energy balance (NEB), prompting adipose tissue to release substantial amounts of NEFA to compensate for the energy deficit. The liver partially oxidizes these NEFA to produce ketone bodies, a process that can lead from lipotoxicity to various metabolic disorders [1]. Elevated NEFA levels are considered one of the primary pathological factors contributing to these metabolic diseases [2]. Extensive research has established a strong correlation between fluctuations in NEFA concentration and metabolic regulation. Particularly, NEFA has been shown to significantly and dose-dependently inhibit the proliferation of peripheral blood mononuclear cells (PBMC) in cows [3]. Furthermore, NEFA influences the mRNA expression levels of molecules associated with lipid metabolism. Specifically, it decreases the expression of molecules involved in lipid catabolism, such as HSL and CPT1A, while increasing the expression of those related to lipid deposition, including FASN and SREBP [4]. Additionally, high concentrations of NEFA have been observed to suppress fatty acid oxidation and up-regulate fatty acid esterification activities in placental trophoblast cells, thereby promoting the formation of lipid droplets [5]. All of these studies have demonstrated the significant impact of elevated NEFA levels on cellular biochemical processes, highlighting their critical role in the pathogenesis of metabolic diseases in cattle.

Against this backdrop, research has gradually shifted towards exploring the role of intracellular key proteins in regulating NEFA metabolism. These key proteins may serve as important targets for counteracting the adverse effects of elevated NEFA levels by participating in biological processes such as fatty acid metabolism, energy balance, oxidative stress, and inflammatory responses. For instance, AMP-activated protein kinase (AMPK), as an energy sensor, can modulate fatty acid oxidation and synthesis pathways, thereby influencing NEFA levels and their metabolic effects [6,7,8]. Additionally, peroxisome proliferator-activated receptors (PPARs), as members of the nuclear receptor family, play a crucial role in fatty acid metabolism and the regulation of insulin sensitivity [9,10]. In light of these findings, investigating whether NDUFAB1 can mitigate the negative effects of high NEFA concentrations represents a promising direction for further research.

The protein NDUFAB1, sometimes referred to as mitochondrial acyl carrier protein, is essential for energy metabolism and cell biology. Previous research has shown a connection between NDUFAB1 and metabolic regulation. For instance, studies have demonstrated that a reduced expression of NDUFAB1 markedly decreases the viability of MCF-7 and MDA-MB-231 cell lines, suggesting its potential role in promoting cell proliferation [11]. Moreover, elevated expression levels of NDUFAB1 have been observed in breast cancer tissues, where high levels of this protein are often associated with poor patient prognosis. In vitro experiments have further corroborated that NDUFAB1 enhances the migration and proliferation of breast cancer cells [11]. In contrast, the overexpression of NDUFAB1 has been shown to confer protective effects against high-fat-diet-induced obesity and insulin resistance in mice [12]. This protective mechanism is attributed to the role of NDUFAB1 in promoting fatty acid oxidation, thereby reducing lipid deposition and preventing obesity associated with excessive lipid deposition. Additionally, NDUFAB1 has been identified as a key gene influencing lipid deposition, further underscoring its importance in lipid metabolism [13,14]. In summary, the findings from these studies collectively indicate a strong association between NDUFAB1 and metabolic regulation.

In accordance with previous research regarding the metabolic regulatory functions of NDUFAB1, this study specifically investigates its role in high-concentration NEFA-induced adipocyte dysfunction in dairy cattle. While current evidence indicates NDUFAB1’s involvement in energy metabolism, its potential protective mechanisms against NEFA-mediated metabolic disorders remain unknown. To address this gap, we systematically investigate how elevated NEFA levels affect adipocyte function while characterizing NDUFAB1’s regulatory role. Our integrated approach aims to elucidate how NEFA exposure modulates NDUFAB1 expression and activity, uncover the molecular mechanisms underlying NDUFAB1’s protective effects against NEFA-induced metabolic dysfunction, and assess its therapeutic potential for bovine metabolic disorders. These findings will provide the first experimental evidence establishing NDUFAB1 as both a key regulator of metabolic homeostasis and a promising therapeutic target for NEFA-associated metabolic dysfunction, advancing our understanding of periparturient metabolic disease pathogenesis.

## 2. Materials and Methods

### 2.1. Gathering and Cultivating Cow Primary Adipocytes

In this study, primary adipocytes from cows were utilized. Under sterile conditions, bovine perirenal adipose tissue was harvested for preadipocyte isolation. The tissue was immersed in PBS containing 4% penicillin–streptomycin in a 6-well plate, and connective tissues and blood vessels were carefully removed. After three washes with PBS, the adipose tissue was enzymatically digested in a 15 mL centrifuge tube containing 0.1% type I collagenase (Gibco, Carlsbad, CA, USA) at 37 °C for 1 h with intermittent agitation every 15 min. The digestion was terminated by adding an equal volume of complete growth medium, followed by sequential filtration through 40 μm and 70 μm cell strainers. The isolated bovine preadipocytes were then seeded into T25 flasks and maintained at 37 °C in a humidified 5% CO_2_ atmosphere, with the medium refreshed every 1–2 days. Cells were routinely passaged upon reaching 70–80% confluence.

### 2.2. Preparation of the NEFA Storage Solution

An NEFA storage solution comprising palmitic, palmitoleic, oleic, linoleic, and stearic acids was first made with a concentration of 52.7 mmol/L. The first step was to prepare a solution of potassium hydroxide (KOH) at 0.1 mmol/L and a solution of hydrochloric acid (HCl) at 1 mol/L. In addition to preheating 113 milliliters of 0.1 mmol/L KOH solution, the water bath’s temperature was set to 60 °C. The KOH solution was then heated, and 0.4097 g of stearic acid, 0.8180 g of palmitic acid, 0.1524 mL of linoleic acid, 1.3752 mL of oleic acid, and 0.1507 mL of palmitoleic acid were added one after the other. After adding 67.8 milliliters of distilled water to the mixture, the solutes were shaken in a water bath to ensure full dissolution. A 1 mol/L HCl solution was then used to bring the solution’s pH down to roughly 7.2. After passing through a 0.22 μm microporous membrane filter, the solution was transferred into 50 mL centrifuge tubes and kept at −20 °C. After passing across a 0.22 μm microporous membrane filter, the solution was transferred into 50 mL centrifuge tubes.

### 2.3. Preparing Sequencing Samples

We conducted RNA-seq analysis on bovine primary adipocytes, including three biological control replicates (CON 1, CON 2, CON 3) and three 0.6 mM NEFA-treated replicates (NEFA 1, NEFA 2, NEFA 3). All sequencing services were provided by Annoroad Gene Technology Co., Ltd. (Beijing, China).

### 2.4. Assay for Cell Viability

We conducted the following tests in accordance with the guidelines supplied by CCK-8 Cell Proliferation and Cytotoxicity Detection Kit (Solarbio, Beijing, China). Initially, 96-well plates were filled with primary cow adipocytes at a density of 1 × 10^3^ cells per well, and the plates were incubated for 24 h at 37 °C with 5% CO_2_. The cells were then treated with 0, 0.2, 0.4, and 0.6 mM NEFA for 0, 12, 24, 36, and 48 h, respectively. Following treatment, each well received 10 μL of CCK-8 solution, and the wells were incubated for an additional two hours at 37 °C. Finally, a Thermo Fisher Scientific (Waltham, MA, USA) spectrophotometer was used to measure each well’s absorbance at 450 nm.

### 2.5. Identification of 5-Ethynyl-2′-deoxyuracil (EDU)

We used the BeyoClick™ EDU Cell Proliferation Kit containing Alexa Fluor 555 labeling (Beyotime, Chengdu, China). Cells were first implanted in 12-well plates, and they were then co-cultured with EdU working solution at a 1:1000 dilution ratio for two hours at 37 °C and 5% CO_2_. After 30 min of fixative treatment, cells were exposed to 0.5% Triton X-100 (CARPRO, Amsterdam, The Netherlands) for 15 min to enhance the permeability of their membranes. Following a 30-min incubation period with Click reaction solution to fluorescently label EDU, cells were subjected to a 20-min DAPI staining procedure to label the nuclei. An Olympus IX73 microscope (Olympus, Tokyo, Japan) was used to obtain fluorescence images, and each pair of samples had at least 12 fields of view captured.

### 2.6. Oil Red O Coloring

First, the cells were fixed in 4% paraformaldehyde for 30 min. Next, oil red O dye (NJBI, Nanjing, China) was mixed with deionized water at a 3:2 ratio before being delivered to the cells and allowed to stand for 15 min to stain. The cells were subsequently washed with phosphate buffer solution (PBS) to remove excess color. Following that, at least three photos were obtained for each experimental group with an inverted microscope (Olympus, Tokyo, Japan). Finally, the absorbance of each sample was measured at 510 nm using an enzyme marker (Thermo Fisher Scientific, Waltham, MA, USA).

### 2.7. Extracting RNA and Quantitative Real-Time PCR

First, the Trizol reagent (Aidlab, Beijing, China) was used to extract the total RNA from cow adipocytes. Then, using a Nanodrop 2000 spectrophotometer (Thermo Fisher Scientific, Waltham, MA, USA), the RNA’s concentration and purity were assessed. The samples were deemed qualified when the RNA’s 260/230 and 260/280 OD values were both more than 1.80. The integrity of the RNA was next evaluated by agarose gel electrophoresis analysis. The integrity of the RNA was further validated by the electrophoresis results, which indicated that the 28S and 18S bands should be distinct and bright. The brightness of the 28S band was roughly double that of the 18S. For backup, qualified RNA samples were kept in a refrigerator set at −80 °C. Following the directions on the mRNA Reverse Transcription Kit (Vazyme, Nanjing, China), the extracted mRNA was then reverse-transcribed. The SYBR^®^ Premix Ex Taq TM reagent (Foregene, Chengdu, China) was then used in real-time quantitative PCR assays using a ForeQuant F4 Sequence Detection System. The following was the setup for the PCR reaction program: one cycle at 95 °C for 10 min was followed by 40 cycles at 95 °C for 10 s each, and finally, at 60 °C for 20 s, when fluorescence signals were recorded. Table 1 provides a detailed list of all primers utilized in this investigation. Gene expression levels were adjusted to beta-actin (β-actin) expression during the data processing stage, and the 2^−ΔΔCt^ method was used to determine each gene’s relative expression levels.

### 2.8. Interference with Synthesis and Transfection

In this investigation, the growth medium was supplemented with Lipofectamine 3000 (Invitrogen, Carlsbad, CA, USA) and Opti-MEM (Gibco, Grand Island, NY, USA) to increase the transfection efficiency. The produced transfection mixes were then put into cultured cell plates and maintained for 48 h to incubate. The sequencing information of the small interfering RNA (SiRNA) and negative control (NC) vectors, which were provided specifically for this research by GenePharma (Shanghai, China), is shown in Table 2.

### 2.9. Protein Extraction and Western Blot

The Western blot technique was employed in this investigation to evaluate the proteins’ expression levels, and β-actin served as an internal reference for standardization control. To effectively separate protein samples, we first used the SDS polyacrylamide gel electrophoresis (SDS-PAGE) technique. The isolated proteins were then placed on a PVDF membrane and given a two-hour confinement procedure. Following confinement, the membrane was incubated with the suitable primary antibody for an entire night at 4 °C (Table 3 lists the antibody dilution concentrations used).

### 2.10. Triglyceride Measurement

Triglyceride (TG) levels in bovine adipocyte samples were measured using a commercial kit (PMK1142, Hubei Pumei Biotechnology Co., Ltd., Jingmen, China). Bovine adipocytes were lysed in extraction buffer provided by the kit, followed by centrifugation at 8000× *g* for 10 min at 4 °C. The supernatant was collected for TG analysis. TG was extracted, hydrolyzed to glycerol, oxidized to formaldehyde, and measured colorimetrically at 420 nm. Concentrations were determined from a standard curve.

### 2.11. RNA Sequencing and Bioinformatics Analysis

RNA samples were subjected to rigorous quality control using an Agilent 2100 Bioanalyzer (Agilent Technologies, Santa Clara, CA, USA). cDNA library preparation was performed using the NEBNext Ultra™ RNA Library Prep Kit for Illumina (New England Biolabs, Ipswich, MA, USA) following the manufacturer’s protocol. Briefly, first-strand cDNA was synthesized using M-MuLV reverse transcriptase, followed by second-strand synthesis with dNTPs and DNA polymerase I. The resulting double-stranded cDNA fragments (250–300 bp) were end-repaired, polyadenylated, and ligated with sequencing adapters prior to PCR amplification. Quality-controlled libraries were pooled based on their effective concentrations and sequencing depth requirements. Paired-end sequencing was performed on an Illumina platform (Illumina Inc., San Diego, CA, USA). Differential gene expression analysis was conducted using DESeq2 (version 1.40.2) with a significance threshold of *p* ≤ 0.05 after the normalization of read counts.

### 2.12. Statistical Analysis

All data are presented as mean ± standard error (SEM). GraphPad 9 software (GraphPad Software Inc., La Jolla, CA, USA) was utilized for statistical analyses, and the Student’s *t*-test was employed to ascertain group differences. Additionally, for multiple comparisons, a one-way ANOVA was employed. The following are the significance levels: * *p* < 0.05, ** *p* < 0.01.

## 3. Results

### 3.1. Effect of Different Treatment Concentrations and Times on Adipocyte Viability

**Figure 1:**
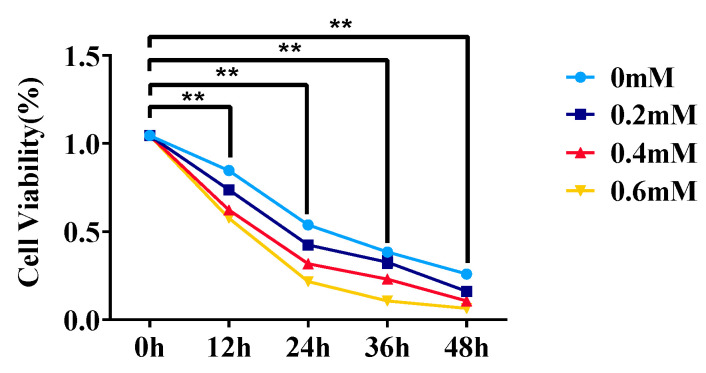
Effects of different treatment concentrations and times on adipocyte viability. Data are expressed as SEM ± mean. ** *p* < 0.01.

CCK-8 was used to measure the cell viability when the cultivated cow adipocytes reached 80% growth. Here, 0, 0.2, 0.4, and 0.6 mM NEFA were used to treat the adipocytes for 0, 12, 24, 36, and 48 h, respectively (Figure 1). According to the results, adipocyte viability declined as NEFA concentration increased, with the most noticeable decrease occurring at 0.6 mM. As a result, 0.6 mM was chosen as the optimal concentration, and 12 h was chosen as the ideal treatment duration based on the impact of varying treatment durations on cow adipocyte viability.

### 3.2. High Concentration of NEFA Inhibits Adipocyte Proliferation

**Figure 2:**
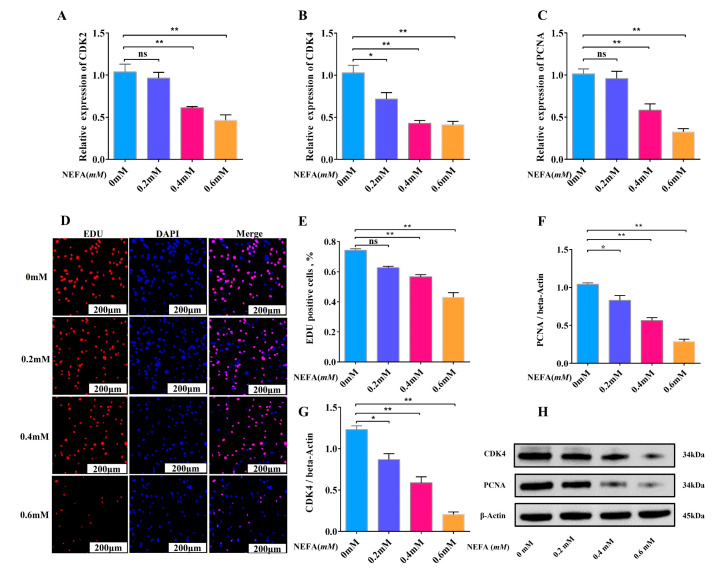
A high concentration of NEFA inhibits the adipocyte proliferation. (**A**–**C**) Relative mRNA expression levels of CDK2, CDK4 and PCNA in adipocytes of NEFA-treated cows (0 mM, 0.2 mM, 0.4 mM, 0.6 mM) (*n* = 6). (**D**) EDU proliferation assay of adipocytes (0 mM, 0.2 mM, 0.4 mM, 0.6 mM) treated with NEFA. Red fluorescence represents EDU-positive cells and blue fluorescence represents DAPI-stained cells. (**E**) Percentage of EDU-positive cells. Rate of EDU-positive cells = EDU-positive cells/DAPI-stained cells × 100%. (*n* = 3). (**F**–**H**) Relative protein expression levels of CDK4 and PCNA in adipocytes of NEFA-treated cows (0 mM, 0.2 mM, 0.4 mM, 0.6 mM) (*n* = 3). Data are expressed as SEM ± mean. * *p* < 0.05; ** *p* < 0.01, ns (no significance).

In order to discover how high NEFA affected adipocyte proliferation, we treated cow adipocytes with 0, 0.2, 0.4, and 0.6 mM NEFA for 12 h. The outcomes of the experiment demonstrate that the amount of NEFA significantly impacted the cows’ adipocyte growth. In particular, we discovered that 0.2 mM NEFA reduced adipocyte proliferation in comparison to the control group, but this difference was not statistically significant. However, when the NEFA concentration was raised to 0.4 mM and 0.6 mM, a notable drop in the expression levels of genes and proteins linked to proliferation was seen in dairy adipocytes (Figure 2A–C,F–H). Furthermore, we discovered that 0.4 mM and 0.6 mM of NEFA could considerably lower the adipocyte proliferation by measuring cell proliferation by EDU (Figure 2D,E). According to the above findings, high NEFA concentrations prevent cattle adipocyte growth by inhibiting the expression of genes linked to proliferation. This study offers a useful resource for learning more about the molecular process underlying NEFA’s impact on cattle adipocyte proliferation.

### 3.3. High Concentration of NEFA Promotes Adipocyte Lipid Deposition

**Figure 3:**
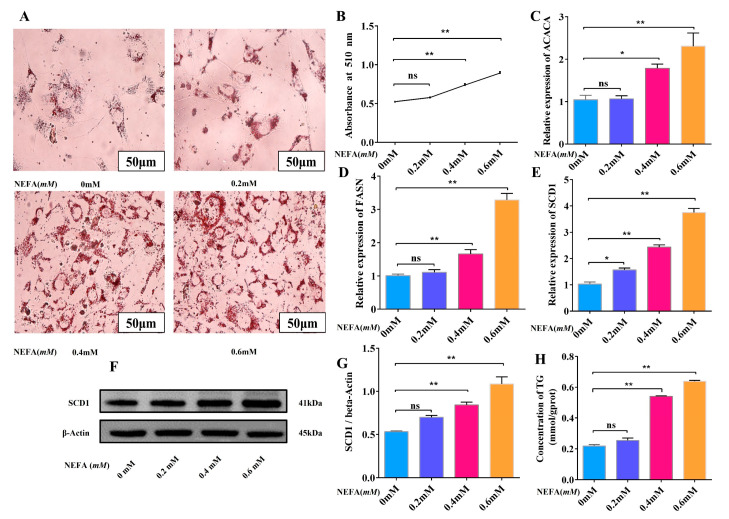
A high concentration of NEFA promotes adipocyte lipid deposition. (**A**,**B**) Oil red O staining of NEFA-treated lipid droplets and absorbance at 510 nm. (**C**–**E**) Relative mRNA expression levels (0 mM, 0.2 mM, 0.4 mM, 0.6 mM) of ACACA, FASN, and SCD1 in adipocytes. (*n* = 6). (**F**–**G**) Relative protein expression levels of SCD1 (0 mM, 0.2 mM, 0.4 mM, 0.6 mM) in adipocytes. (*n* = 3). (**H**) TG content after NEFA treatment. Data are expressed as SEM ± mean. * *p* < 0.05; ** *p* < 0.01, ns (no significance).

In this study, we treated cow adipocytes with 0, 0.2, 0.4, and 0.6 mM NEFA for 12 h to see how it affected the adipocytes’ lipid deposition. The results show that the number of intracellular lipid droplets gradually increased with increasing NEFA concentration (Figure 3A,B). In addition, RT-qPCR analysis revealed that 0.2 mM NEFA treatment did not significantly affect adipocyte viability compared to the control group. However, when the NEFA concentration was increased to 0.4 mM and 0.6 mM, a marked elevation in intracellular lipid deposition was observed in adipocytes, a result that is consistent with oil red O staining (Figure 3C–E). In contrast, WB analysis demonstrated that NEFA could significantly promote lipid deposition even at lower concentrations (Figure 3F,G). In addition, the triglyceride findings show a gradual increase in TG with increasing NEFA concentration (Figure 3H). This discrepancy between RT-qPCR and WB results suggests that these two experimental approaches may elucidate distinct aspects of NEFA’s regulatory mechanisms on lipid metabolism in adipocytes. According to these findings, adipocytes were lipotoxic to high concentrations of NEFA, which also encouraged fat buildup and abnormalities of lipid metabolism, posing health risks.

### 3.4. Construction of a Differentiation Model and Inhibition of Adipocyte Differentiation by the Optimal Concentration of NEFA

**Figure 4:**
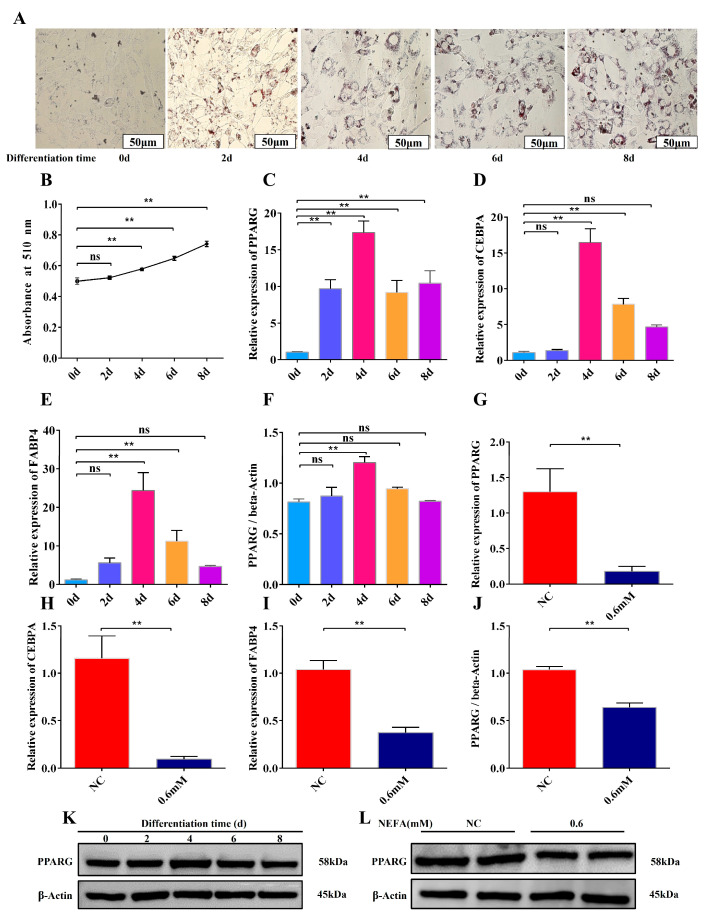
Construction of a differentiation model and inhibition of adipocyte differentiation by optimal concentration of NEFA. (**A**,**B**) Oil red O staining of lipid droplets at 0, 2, 4, 6 and 8 d of differentiation and absorbance at 510 nm. (**C**–**E**) Relative mRNA expression levels of PPARG, CEBPA and FABP4 during adipocyte differentiation in dairy cows (*n* = 6). (**F**–**K**) Relative protein expression levels of PPAR during adipocyte differentiation in dairy cows (*n* = 3). (**G**–**I**) Relative mRNA expression levels of PPARG, CEBPA and FABP4 in cow adipocytes treated with 0.6 mM NEFA at d 4 of differentiation (*n* = 6). (**J**–**L**) Relative protein expression levels of PPARG in adipocytes from cows treated with 0.6 mM NEFA at 4 d of differentiation (*n* = 3). Data are expressed as SEM ± mean. ** *p* < 0.01, ns (no significance).

In our research, we successfully modeled the differentiation of adipocytes in dairy cows using oil red O staining and performed absorbance measurements. The findings demonstrate that as the period of differentiation days increased, the amounts of intracellular lipid droplets increased gradually (Figure 4A,B). Furthermore, using RT-qPCR and WB assay analysis, we discovered that the expression levels of the differentiation-associated protein PPARG (peroxisome proliferator-activated receptor G), as well as the important differentiation-related genes C/EBPA (enhancer-binding protein), FABP4 (fatty acid-binding protein 4), and PPARG, increased gradually during the early stages of differentiation (days 0–4), and then decreased during the middle and late stages (6–8 days). Particularly, these genes and proteins reached peak expression at day 4 of differentiation (Figure 4C–F,K). As a result, we determined that day 4 was the ideal time point for differentiation. We then treated adipocytes with the ideal concentration (0.6 mM) of NEFA for 12 h after they had differentiated to day 4 in order to investigate its impact on the differentiation process. According to the experimental findings, adipocyte differentiation was inhibited by 0.6 mM NEFA treatment, which also markedly decreased the expression levels of genes and proteins linked to differentiation (Figure 4G–L). According to this research, bovine adipocyte differentiation may be negatively regulated by excessive NEFA concentrations.

### 3.5. Analysis of Transcriptomics Sequencing Results

**Figure 5:**
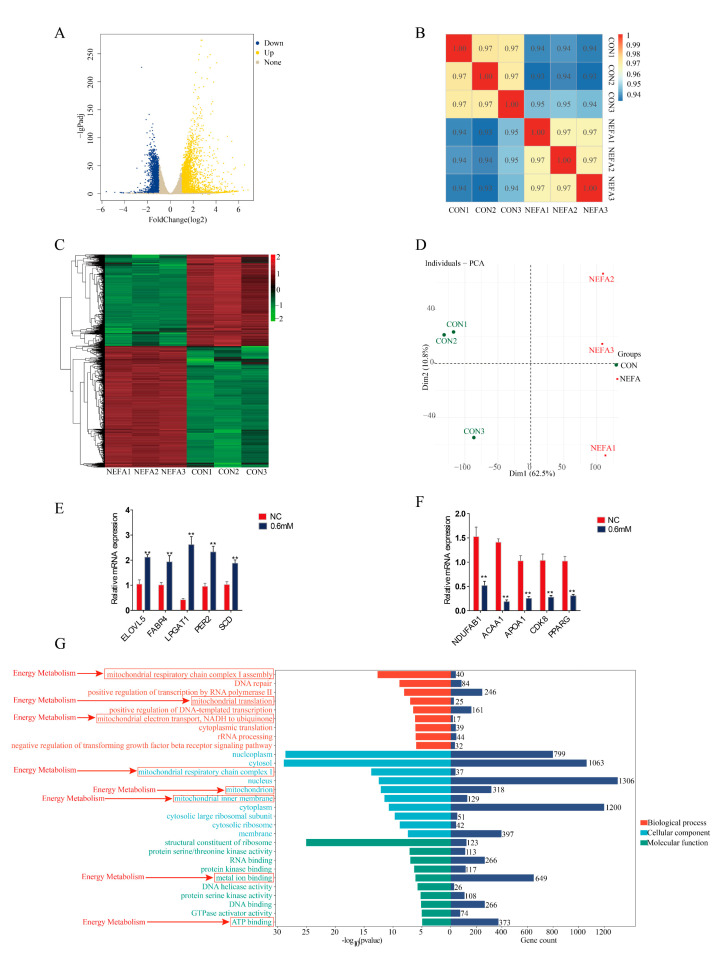
Analysis of differentially expressed genes. (**A**) Volcano plot of differentially expressed genes between control (CON group) and 0.6 mM NEFA-treated group. (**B**) Correlation heatmap of differentially expressed genes between control (CON group) and 0.6 mM NEFA treatment group. (**C**,**D**) Heatmaps of differentially expressed genes between the control group (CON group) and the 0.6 mM NEFA-treated group with principal component analysis. (**E**,**F**) RT-qPCR validation of 10 differentially expressed genes. (**G**) Bar graph of GO enrichment of differential genes between control (CON group) and 0.6 mM NEFA-treated group. Data are expressed as SEM ± mean. ** *p* < 0.01.

Transcriptomic profiling revealed 4638 differentially expressed genes (DEGs) between NEFA-treated and control (CON) groups, including 2642 up-regulated and 1996 down-regulated genes (Figure 5A). Quality assessment demonstrated strong intra-group reproducibility (r > 0.94) and significant inter-group differences (r < 0.65), confirming distinct transcriptional profiles (Figure 5B). Additionally, multivariate analysis showed clear separation between groups, with principal component analysis (PCA) revealing 62.5% variance along PC1 (Figure 5C) and hierarchical clustering demonstrating distinct expression patterns (Figure 5D). The validation of the 20 selected DEGs by RT-qPCR showed high concordance with sequencing data (Figure 5E,F). Subsequently, functional analysis identified NDUFAB1 as the most significantly down-regulated gene. GO enrichment revealed significant alterations in mitochondrial energy metabolism pathways, including mitochondrial respiratory chain complex I assembly, ATP binding and metal ion binding (Figure 5G). These findings suggest that NEFA-induced NDUFAB1 downregulation disrupts mitochondrial function, potentially explaining NEFA-mediated adipocyte dysfunction through impaired energy metabolism and lipid deposition.

### 3.6. Interference with NDUFAB1 and NEFA Co-Treatment Inhibits Adipocyte Proliferation

**Figure 6:**
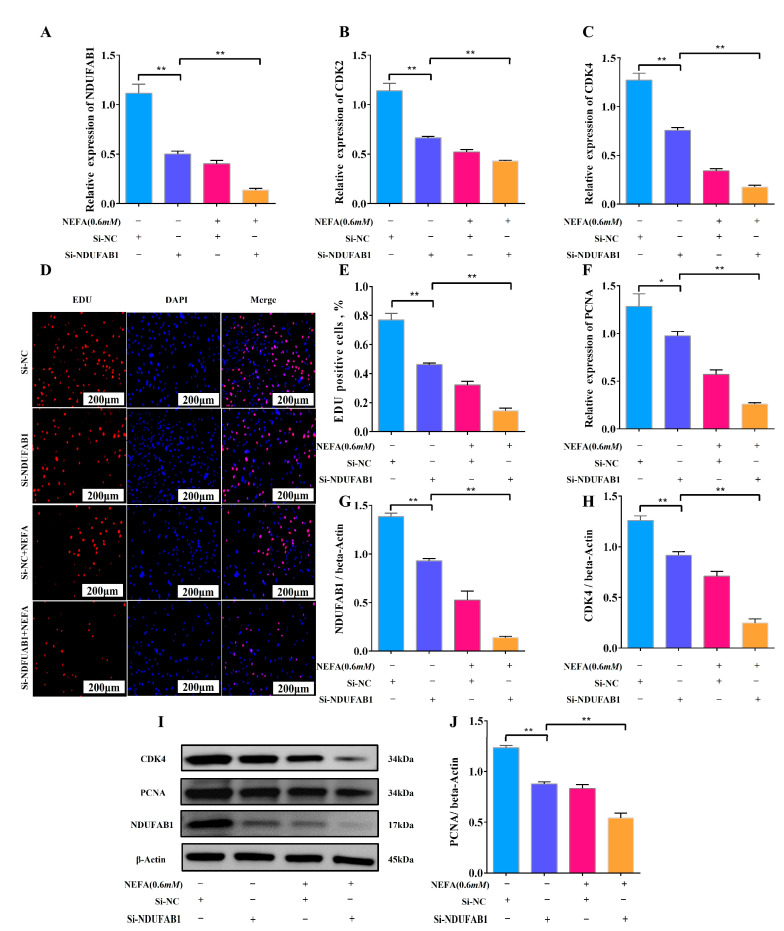
Interference with NDUFAB1 and NEFA co-treatment inhibits adipocyte proliferation. (**A**–**C**,**F**) Relative mRNA expression levels of NDUFAB1, CDK2, CDK4 and PCNA in cow adipocytes after transfection of Si-NC with si-NDUFAB1 and co-treatment (*n* = 6). (**D**) EDU proliferation assay after transfection of Si-NC with Si-NDUFAB1 and co-treatment. Red fluorescence represents EDU-positive cells and blue fluorescence represents DAPI-stained cells. (**E**) Percentage of EDU-positive cells after transfection of Si-NC with Si-NDUFAB1 and after co-treatment. Rate of EDU-positive cells = EDU-positive cells/DAPI-stained cells × 100%. (*n* = 3). (**G**–**J**) Relative protein expression levels of NDUFAB1, CDK4, and PCNA in adipocytes from cows after transfection of Si-NC with si-NDUFAB1 and co-treatment (*n* = 3). Data are expressed as SEM ± mean. * *p* < 0.05; ** *p* < 0.01.

To evaluate the influence of the differentially expressed gene NDUFAB1 on bovine adipocyte proliferation, our research used a combination of Si-NDUFAB1 and NEFA for verification. The experimental results reveal that Si-NDUFAB1 alone significantly reduced the relative mRNA expression of cell cycle regulation-related genes CDK2, CDK4, and PCNA compared to the control group, and this inhibition of proliferation was exacerbated when Si-NDUFAB1 was co-treated with NEFA (Figure 6A–C,F). Furthermore, the results obtained by WB were similar to the mRNA expression levels shown above, confirming the inhibitory effects of Si-NDUFAB1 and co-treatment on adipocyte proliferation (Figure 6G–J). In addition, we investigated its effect on cell viability using EDU, and the results show that Si-NDUFAB1 alone and in combination with NEFA significantly decreased adipocyte viability, limiting adipocyte proliferation (Figure 6D,E). These findings imply that NDUFAB1 may enhance cattle adipocyte proliferation and may also serve as a therapeutic target for NEFA-induced adipocyte metabolic abnormalities.

### 3.7. Interference with NDUFAB1 and NEFA Co-Treatment Promotes Lipid Deposition

**Figure 7:**
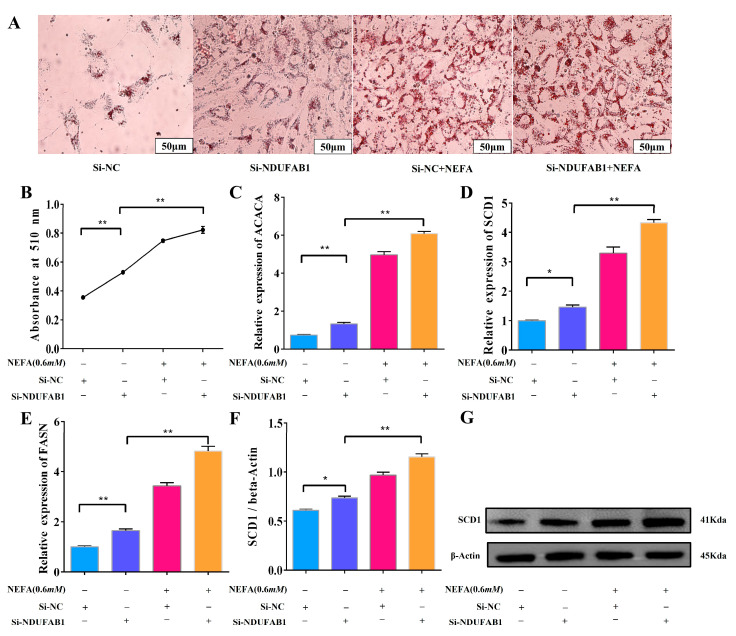
Interference with NDUFAB1 and NEFA co-treatment promotes lipid deposition. (**A**,**B**) Oil red O staining and absorbance at 510 nm of lipid droplets after transfection of Si-NC with Si-NDUFAB1 and co-treatment. (**C**–**E**) Relative mRNA expression levels of ACACA, FASN and SCD1 in cow adipocytes after transfection of Si-NC with Si-NDUFAB1 and co-treatment (*n* = 6). (**F**–**G**) Relative protein expression levels of SCD1 in adipocytes of cows transfected with Si-NC versus Si-NDUFAB1 and after co-treatment (*n* = 3). Data are expressed as SEM ± mean. * *p* < 0.05; ** *p* < 0.01.

In order to investigate the specific effects of the differentially expressed gene NDUFAB1 on lipid deposition in bovine adipocytes, oil red O staining as well as the co-treatment of Si-NDUFAB1 with NEFA were used in the present study to explore its effects on lipid deposition. When Si-NDUFAB1-treated adipocytes were compared to the control group, the number of lipid droplets increased significantly, and when Si-NDUFAB1 was co-treated with NEFA, the number of lipid droplets increased even more (Figure 7A,B). These results suggest that NDUFAB1 may have an inhibitory effect on lipid deposition. In addition, Si-NDUFAB1 was found to be able to significantly raise the relative mRNA expression levels of the genes related to lipid deposition, including FASN, ACACA and SCD1. This up-regulation effect was more noticeable following the co-treatment of Si-NDUFAB1 with NEFA, indicating that the co-treatment further promoted lipid deposition (Figure 7C–E). Additionally, WB was used to get results that were in line with RT-qPCR (Figure 7F,G). By speeding up the oxidation of fatty acids, NDUFAB1 may effectively prevent lipid deposition in adipocytes, according to the results above. At the same time, it might also lessen the harm that high NEFA concentrations bring to cattle adipocytes through lipotoxicity.

### 3.8. Interference with NDUFAB1 and NEFA Co-Treatment Inhibits Adipocyte Differentiation

**Figure 8:**
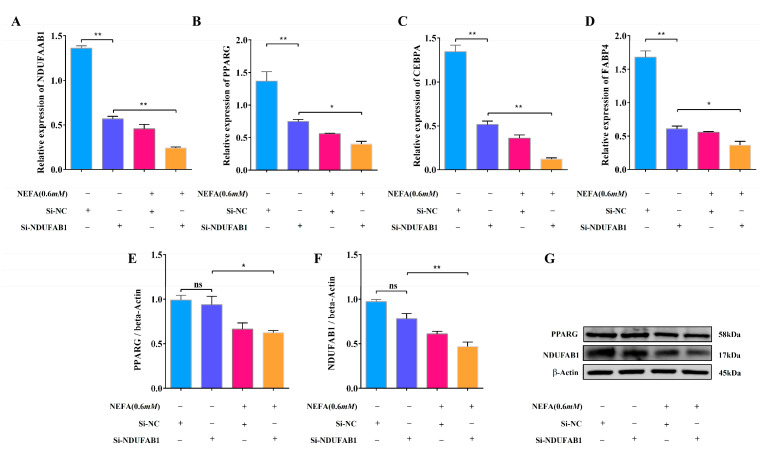
Interference with NDUFAB1 and NEFA co-treatment inhibits adipocyte differentiation. (**A**–**D**) Relative mRNA expression levels of NDUFAB1, PPARG, CEBPA, and FABP4 in adipocytes of cows transfected with Si-NC versus Si-NDUFAB1 and after co-treatment (*n* = 6). (**E**–**G**) Relative protein expression levels of NDUFAB1 and PPARG in adipocytes of cows transfected with Si-NC versus Si-NDUFAB1 and after co-treatment (*n* = 3). Data are expressed as SEM ± mean. * *p* < 0.05; ** *p* < 0.01, ns (no significance).

In order to investigate the effect of the differentially expressed gene NDUFAB1 on bovine adipocyte differentiation, the combined treatment of Si-NDUFAB1 and NEFA was used in this study. The findings reveal that Si-NDUFAB1 alone significantly reduced the relative mRNA expressions of differentiation marker genes PPARG, CEBPA, and FABP4 in adipocytes compared to the control group. The mRNA expression levels of these differentiation marker genes were significantly down-regulated when Si-NDUFAB1 was co-treated with NEFA, indicating that the co-treatment’s inhibitory effect on adipocyte development was even more significant (Figure 8A–D). Furthermore, Western blot experiments confirmed that Si-NDUFAB1 treatment alone reduced PPAR protein expression, while co-treatment significantly reduced differentiation-related protein expression (Figure 8E–G). The study’s findings imply that NDUFAB1 may have the ability to promote adipocyte differentiation, and by controlling adipocyte differentiation, may slow down the metabolic disorders brought on by high NEFA concentrations. This makes it a possible therapeutic target for preventing metabolic diseases in cows.

## 4. Discussion

NEFA plays a critical role in metabolic regulation and pathophysiology, particularly in dairy cows during periods of metabolic stress. It has been well-documented that elevated NEFA levels, such as 0.6 mM, significantly impair hepatocyte viability and promote lipid deposition, leading to lipotoxicity [4]. Consistent with these findings, our research also demonstrated that 0.6 mM NEFA led to lipid deposition and reduced cell viability. Notably, NEFA concentrations exceeding 0.4 mmol/L are recognized as a biomarker of enhanced lipid mobilization and negative energy balance in dairy cows, particularly during the periparturient period [15]. This metabolic imbalance often disrupts the equilibrium between fatty acid synthesis and oxidation in the liver, resulting in the progressive accumulation of triglycerides (TAG) and exacerbating the development of ketosis [16]. Beyond hepatic effects, elevated NEFA levels have been shown to exert dose-dependent inhibitory effects on the proliferation of bovine mammary epithelial cells (BMECs), accompanied by the activation of inflammatory pathways [17]. In cows with clinical ketosis, increased NEFA levels significantly upregulated the expression and activity of hepatic cytochrome P450 2E1 (CYP2E1), contributing to oxidative stress and further metabolic dysregulation [18]. Additionally, high NEFA concentrations have been found to induce endoplasmic reticulum (ER) stress, trigger the unfolded protein response (UPR), and promote inflammatory signaling pathways [19]. During periods of energy imbalance, excessive NEFA accumulation in ovarian tissue and preantral follicles has been shown to alter the expression of ovarian-related genes, potentially compromising reproductive function [20]. Additionally, NEFA has been demonstrated to elevate intracellular reactive oxygen species (ROS) levels through the activation of the Nrf2/p53 signaling pathway, leading to endoplasmic reticulum stress (ERS) and subsequent apoptosis in granulosa cells [21]. The molecular mechanisms underlying NEFA-induced cellular dysfunction may involve the activation of the PI3K/AKT/FoxO1 signaling pathway, which regulates proliferation and apoptosis in bovine granulosa cells [22]. The role of NEFA in inflammatory responses is further underscored by its ability to enhance the production of inflammatory cytokines in ketosis cow neutrophils through the activation of the TLR2/4-NF-κB signaling pathway [23]. Furthermore, NEFA has been shown to directly influence lipid metabolism by stimulating lipid deposition and oxidation in bovine hepatocytes via the PERK-eIF2α signaling pathway, while simultaneously inhibiting the formation and secretion of very-low-density lipoprotein (VLDL) [24]. Furthermore, in calf hepatocytes, NEFA activates the NF-κB signaling pathway, triggering inflammatory responses that may disrupt normal cellular differentiation and function [25]. Our findings are consistent with previous research, and we have elucidated for the first time the inhibitory effects of high NEFA levels on bovine adipocyte proliferation and differentiation. Furthermore, we discovered that lipid deposition was dose-dependently enhanced by elevated NEFA levels. These results provided a crucial foundation for a more thorough comprehension of NEFA’s function in the pathophysiology.

NDUFAB1, a mitochondrial acyl carrier protein and integral component of mitochondrial respiratory chain complex I, plays a pivotal role in electron transfer, and is essential for maintaining mitochondrial energy metabolism [26,27]. As the primary location of energy metabolism in adipocytes, mitochondria are directly impacted by variations in NDUFAB1 expression in terms of their bioenergetic state and the metabolism of reactive oxygen species. An essential energy source for cell growth and development as well as the production of fatty acids, ATP is produced in the mitochondrial respiratory chain via electron transfer and the creation of a proton gradient [28]. In this study, transcriptomic analysis revealed that elevated NEFA levels might disrupt ATP synthesis by impairing NDUFAB1 function, thereby compromising mitochondrial integrity and leading to metabolic imbalances in adipocytes. This disruption ultimately hindered fatty acid synthesis, cell proliferation, and differentiation. The critical role of NDUFAB1 in metabolic regulation has been well-documented. For instance, it has been demonstrated that NDUFAB1 promotes granulosa cell proliferation, suppresses apoptosis, and enhances steroidogenesis by upregulating the expression of CCND1, BCL-2, STAR, and CYP11A1, while downregulating caspase-3 expression [29]. Furthermore, as a critical regulator of mitochondrial fatty acid synthesis, NDUFAB1 plays an essential role in lipid metabolism pathways. Previous studies have demonstrated that the overexpression of NDUFAB1 enhances the lipoylation of pyruvate dehydrogenase (PDH), thereby increasing PDH activity and promoting fatty acid oxidation. In contrast, a deficiency of NDUFAB1 has been shown to inhibit PDH lipoylation, reduce PDH activity, and impair the metabolic conversion of pyruvate to acetyl-coenzyme A, highlighting its pivotal role in maintaining mitochondrial metabolic homeostasis. These findings underscore the importance of NDUFAB1 in regulating lipid metabolism and energy production, providing a mechanistic basis for its involvement in cellular metabolic processes [30]. These results imply that NDUFAB1 has a possible antagonistic effect on NEFA and somewhat stimulates fatty acid oxidation.

The findings of our study collectively highlight the multifaceted role of NEFA in metabolic dysregulation, and provide novel insights into the molecular mechanisms underlying NEFA-induced cellular dysfunction. Our findings demonstrate that elevated NEFA levels impair adipocyte proliferation (Figure 2) and differentiation (Figure 4) while promoting lipid deposition (Figure 3). The inhibition of NDUFAB1 under high NEFA conditions likely contributed to metabolic imbalances, including impaired ATP synthesis, reduced fatty acid oxidation, and increased lipid deposition, consistent with previous studies showing NEFA-induced mitochondrial dysfunction and lipotoxicity [4,16,24]. The antagonistic relationship between NDUFAB1 and NEFA suggests that NDUFAB1 serves as a protective factor against NEFA-induced metabolic disturbances by enhancing fatty acid oxidation (Figure 7), promoting cell proliferation (Figure 6) and differentiation (Figure 8). This aligns with earlier reports demonstrating NDUFAB1’s role in promoting metabolic regulation in other cell types [29,30]. While these in vitro findings establish NDUFAB1 as a central regulator of NEFA-mediated metabolic dysfunction, several considerations warrant attention. First, the absence of systemic factors and tissue-level interactions in our adipocyte model may limit direct translation to in vivo physiology. Second, the complex interplay between NDUFAB1 and other metabolic pathways, particularly inflammatory signaling networks, remains to be fully elucidated. Future studies employing animal models, human clinical samples, and multi-omics approaches will be crucial to validate these mechanisms in physiological contexts and explore therapeutic opportunities. Such investigations should particularly address the challenges of targeted NDUFAB1 modulation and the cross-species conservation of these metabolic relationships.

## 5. Conclusions

In conclusion, our study demonstrates that elevated NEFA concentrations impair bovine adipocyte metabolism by suppressing NDUFAB1 expression, identifying it as a key regulator of metabolic homeostasis and a potential therapeutic target for NEFA-induced dysfunction. Future studies should elucidate NDUFAB1’s mechanistic role in mitochondrial metabolism and evaluate its in vivo therapeutic potential, particularly its interactions with inflammatory and stress pathways. These findings provide new opportunities for developing interventions against metabolic disorders in dairy cattle and related species.

## Figures and Tables

**Table 1:** Primer sequences.

Primer Name	Sequence
CDK2-F	AAGCCAGAAACAAGTTGACGG
CDK2-R	TTGATGAGGGGAAGAGGAATG
CDK4-F	CTTTGACCTGATTGGACTGCC
CDK4-R	CACTCTGCGTCACCTTCTGC
PCNA-F	CTTGAAGAAAGTGCTGGAGGC
PCNA-R	TTGGACATGCTGGTGAGGTT
PPARG-F	AGCATTTCCACTCCGCACTA
PPARG-R	GGGATACAGGCTCCACTTTGAT
C/EBPA-F	CGGCAACGACTTTGACTACC
C/EBPA-R	TGCTTCGCTTCGTCCTCCTC
FABP4-F	TTTGAATGGGGGTGTGGTCA
FABP4-R	ACGATGCTCTTGACTTTCCTGT
SCD1-F	ACCTGGCTGGTGAATAGTGC
SCD1-R	TCATAAGCCAGACCGATGGC
ACACA-F	CTGGCTGGACAGACTGATAGCA
ACACA-R	CAACTCCCAGCATGGTGTCA
FASN-F	CGAGTCCCCTGACCACTATCTG
FASN-R	AGGCGTGTGCTCCATGTTCT
β-Actin-F	GCCCATCTATGAGGGGTACGC
β-Actin-R	CTCCTTGATGTCACGGACGATTTC
ELOVL5-F	CGTCCCCACTTTGGTCTGTT
ELOVL5-R	CCTTCCCATACTCCCGTCAC
LPGAT1-F	GGCTTCTTGGGGATGGTATG
LPGAT1-R	TCTCCTGTTGCCTGATGGTTC
PER2-F	ATGTCCGCCTACATTACCGAG
PER2-R	TTTCCAACCTGTGACCCTTCT
NDUFAB1-F	GTGCCTGGAACAGTCACACA
NDUFAB1-R	ACTCGGTCCTTGATTCCGTC
ACAA1-F	ACGACAAGGGCACAGAGCA
ACAA1-R	GAGTTTCCAGCCGTGGTAGAG
APOA1-F	ACCTTGGCTGTGCTCTTCCT
APOA1-R	CACGGTGGCAAAATCCTTC

Note: Primer Name: The identifier for each primer, indicating the gene target and whether it is the forward (F) or reverse (R) strand. Sequence: The nucleotide sequence of each primer.

**Table 2:** Sequences of forward and reverse siRNA oligonucleotides targeting NDUFAB1.

Name	Primer Sequences
Si-NDUFAB1-F	CAGGUUCCAGGCAGAGUUA
Si-NDUFAB1-R	UAACUCUGCCUGGAACCUG

Note: siRNA sequences (5′→3′) targeting Bos taurus NDUFAB1 (NCBI NM_001040488). Name: The identifier for each siRNA oligonucleotide, indicating whether it is the forward (F) or reverse (R) strand. Primer Sequences: The nucleotide sequences of the siRNA oligonucleotides.

**Table 3:** Antibody dilution concentration.

Antibody	Company	Accession Number	Dilution Ratio
anti-CDK4	Zenbio (Chengdu, China)	R23886	1:3000
anti-PCNA	Zenbio (Chengdu, China)	R25293	1:3000
anti-SCD1	GenuIN Biotech (Pingdingshan, China)	#51311	1:1000
anti-PPARG	Nature Biosciences (Shanghai, China)	A72166	1:1000
anti-NDUFAB1	ABclonal (Wuhan, China)	A2114	1:500
anti-β-Actin	GenuIN (Hefei, China)	#2885	1:5000

Note: Antibody: The name of the specific antibody used in the experiments. Company: The supplier of the antibody. Accession Number: The unique identifier provided by the supplier for the specific antibody product. Dilution Ratio: The recommended dilution ratio for the antibody as used in the experiments.

## Data Availability

The original western blot images presented in this study are available in Supplementary Materials.

## Supplementary Materials

The following supporting information can be downloaded at: https://www.mdpi.com/article/10.3390/ani15111618/s1. File S1. Original western blot images.

## Abbreviations

ACAA1	Acetyl-CoA Acyltransferase 1
ACACA	Acetyl-CoA Carboxylase Alpha
APOA1	Apolipoprotein A1
CDK2	Cyclin-Dependent Kinase 2
CDK4	Cyclin-Dependent Kinase 4
C/EBPα	CCAAT/Enhancer Binding Protein Alpha
ELOV5	ELOVL Fatty Acid Elongase 5
FABP4	Fatty Acid Binding Protein 4
FASN	Fatty Acid Synthase
LPGAT1	Lysophosphatidylglycerol Acyltransferase 1
NEFA	Non-Esterified Fatty Acids
NDUFAB1	NADH: Ubiquinone Oxidoreductase Subunit AB1
PCNA	Proliferating Cell Nuclear Antigen
PER2	Period Circadian Regulator 2
PPARG	Peroxisome Proliferator Activated Receptor Gamma
SCD	Stearoyl-CoA Desaturase
TG	Triglycerides
β-actin	Beta-Actin

## References

[1] Melendez P., Serrano M.V. (2024). Update on ketosis in dairy cattle with major emphasis on subclinical ketosis and abdominal adiposity. Vet. Med. Sci..

[2] Zhu Y., Liu G., Du X., Shi Z., Jin M., Sha X., Li X., Wang Z., Li X. (2019). Expression patterns of hepatic genes involved in lipid metabolism in cows with subclinical or clinical ketosis. J. Dairy Sci..

[3] Vanacker N., Blouin R., Ster C., Lacasse P. (2022). Effect of different fatty acids on the proliferation and cytokine production of dairy cow peripheral blood mononuclear cells. J. Dairy Sci..

[4] Fan X., Xu J., Hu Y., Wang K., Zhao Y., Cai J., Zhang X., Pan B., Xu A., Chen Y. (2024). Effect of high NEFA concentration on lipid metabolism disorders in hepatocytes based on lipidomics. Front. Pharmacol..

[5] Pathmaperuma A.N., Mana P., Cheung S.N., Kugathas K., Josiah A., Koina M.E., Broomfield A., Delghingaro-Augusto V., Ellwood D.A., Dahlstrom J.E. (2010). Fatty acids alter glycerolipid metabolism and induce lipid droplet formation, syncytialisation and cytokine production in human trophoblasts with minimal glucose effect or interaction. Placenta.

[6] Liu P., Wang L., Wang Y., Jin L., Gong H., Fan J., Zhang Y., Li H., Fu B., Wang Q. (2024). ANXA1-FPR2 axis mitigates the susceptibility to atrial fibrillation in obesity via rescuing AMPK activity in response to lipid overload. Cardiovasc. Diabetol..

[7] Ozcan C., Dixit G., Li Z. (2021). Activation of AMP-Activated Protein Kinases Prevents Atrial Fibrillation. J. Cardiovasc. Transl. Res..

[8] Zhang Y., Fu Y., Jiang T., Liu B., Sun H., Zhang Y., Fan B., Li X., Qin X., Zheng Q. (2021). Enhancing Fatty Acids Oxidation via L-Carnitine Attenuates Obesity-Related Atrial Fibrillation and Structural Remodeling by Activating AMPK Signaling and Alleviating Cardiac Lipotoxicity. Front. Pharmacol..

[9] Zhang L., Yang C., Ding X., Zhang H., Luan Y., Tang Y., Liu Z. (2024). Berberine Ameliorates High-fat-induced Insulin Resistance in HepG2 Cells by Modulating PPARs Signaling Pathway. Curr. Comput. Aided Drug Des..

[10] Li W., Wang Y., Liu C., Yu Y., Xu L., Dong B. (2025). Evaluation of the Regulatory Effect of the Pan-PPAR Agonist Chiglitazar on the Dawn Phenomenon. Diabetes Ther. Res. Treat. Educ. Diabetes Relat. Disord..

[11] Chen Y., Wu W., Jin C., Cui J., Diao Y., Wang R., Xu R., Yao Z., Li X. (2023). Integrating Single-Cell RNA-Seq and Bulk RNA-Seq Data to Explore the Key Role of Fatty Acid Metabolism in Breast Cancer. Int. J. Mol. Sci..

[12] Zhang R., Hou T., Cheng H., Wang X. (2019). NDUFAB1 protects against obesity and insulin resistance by enhancing mitochondrial metabolism. FASEB J. Off. Publ. Fed. Am. Soc. Exp. Biol..

[13] Zhu J., Wang Y., Su Y., Zheng M., Cui H., Chen Z. (2024). RNA sequencing identifies key genes involved in intramuscular fat deposition in chickens at different developmental stages. BMC Genom..

[14] Chang X., Xing P. (2022). Identification of a novel lipid metabolism-related gene signature within the tumour immune microenvironment for breast cancer. Lipids Health Dis..

[15] González F.D., Muiño R., Pereira V., Campos R., Benedito J.L. (2011). Relationship among blood indicators of lipomobilization and hepatic function during early lactation in high-yielding dairy cows. J. Vet. Sci..

[16] Ferré P., Foufelle F. (2010). Hepatic steatosis: A role for de novo lipogenesis and the transcription factor SREBP-1c. Diabetes Obes. Metab..

[17] Li M., Wang H., Ren H., Zhang T., Zhou G., Chen S., Wang J., Jia X., Lai S., Gan X. (2024). L-Histidine attenuates NEFA-induced inflammatory responses by suppressing Gab2 expression. Life Sci..

[18] Peng Z., Li X., Wang Z., Liu G., Li X. (2019). The effects of non-esterified fatty acids and β-hydroxybutyrate on the hepatic CYP2E1 in cows with clinical ketosis. J. Dairy Res..

[19] Yan Y., Huang J., Huan C., Li L., Li C. (2022). Non-Esterified Fatty Acid Induces ER Stress-Mediated Apoptosis via ROS/MAPK Signaling Pathway in Bovine Mammary Epithelial Cells. Metabolites.

[20] Pedroza G.H., Lanzon L.F., Rabaglino M.B., Walker W.L., Vahmani P., Denicol A.C. (2022). Exposure to non-esterified fatty acids in vitro results in changes in the ovarian and follicular environment in cattle. Anim. Reprod. Sci..

[21] Wang Y., Li C., Li J., Wang G., Li L. (2020). Non-Esterified Fatty Acid-Induced Reactive Oxygen Species Mediated Granulosa Cells Apoptosis Is Regulated by Nrf2/p53 Signaling Pathway. Antioxidants.

[22] Lei Z., Ali I., Yang M., Yang C., Li Y., Li L. (2023). Non-Esterified Fatty Acid-Induced Apoptosis in Bovine Granulosa Cells via ROS-Activated PI3K/AKT/FoxO1 Pathway. Antioxidants.

[23] Zhang Y., Li X., Zhang H., Zhao Z., Peng Z., Wang Z., Liu G., Li X. (2018). Non-Esterified Fatty Acids Over-Activate the TLR2/4-NF-Κb Signaling Pathway to Increase Inflammatory Cytokine Synthesis in Neutrophils from Ketotic Cows. Cell. Physiol. Biochem. Int. J. Exp. Cell. Physiol. Biochem. Pharmacol..

[24] Huang Y., Zhao C., Kong Y., Tan P., Liu S., Liu Y., Zeng F., Yuan Y., Zhao B., Wang J. (2021). Elucidation of the mechanism of NEFA-induced PERK-eIF2α signaling pathway regulation of lipid metabolism in bovine hepatocytes. J. Steroid Biochem. Mol. Biol..

[25] Shi X., Li D., Deng Q., Li Y., Sun G., Yuan X., Song Y., Wang Z., Li X., Li X. (2015). NEFAs activate the oxidative stress-mediated NF-κB signaling pathway to induce inflammatory response in calf hepatocytes. J. Steroid Biochem. Mol. Biol..

[26] Hou T., Zhang R., Jian C., Ding W., Wang Y., Ling S., Ma Q., Hu X., Cheng H., Wang X. (2019). NDUFAB1 confers cardio-protection by enhancing mitochondrial bioenergetics through coordination of respiratory complex and supercomplex assembly. Cell Res..

[27] Triepels R., Smeitink J., Loeffen J.L.C.M., Smeets R., Buskens C., Trijbels F., van den Heuvel L.P.W.J. (1999). The human nuclear-encoded acyl carrier subunit (NDUFAB1) of the mitochondrial complex I in human pathology. J. Inherit. Metab. Dis..

[28] Ryu K.W., Fung T.S., Baker D.C., Saoi M., Park J., Febres-Aldana C.A., Aly R.G., Cui R., Sharma A., Fu Y. (2024). Cellular ATP demand creates metabolically distinct subpopulations of mitochondria. Nature.

[29] Sun X., Chen X., Zhao J., Ma C., Yan C., Liswaniso S., Xu R., Qin N. (2021). Transcriptome comparative analysis of ovarian follicles reveals the key genes and signaling pathways implicated in hen egg production. BMC Genom..

[30] Lambrechts R.A., Schepers H., Yu Y., van der Zwaag M., Autio K.J., Vieira-Lara M.A., Bakker B.M., Tijssen M.A., Hayflick S.J., Grzeschik N.A. (2019). CoA-dependent activation of mitochondrial acyl carrier protein links four neurodegenerative diseases. EMBO Mol. Med..

