# Supplementary material for: NDUFAB1 as a Novel Regulator of NEFA-Induced Metabolic Dysfunction in Bovine Adipocytes
Source: Animals (Basel). 2025 May 30;15(11):1618. doi: 10.3390/ani15111618 (PMC12153833; doi:10.3390/ani15111618)

Figure S1. WB original image of Figure 2H

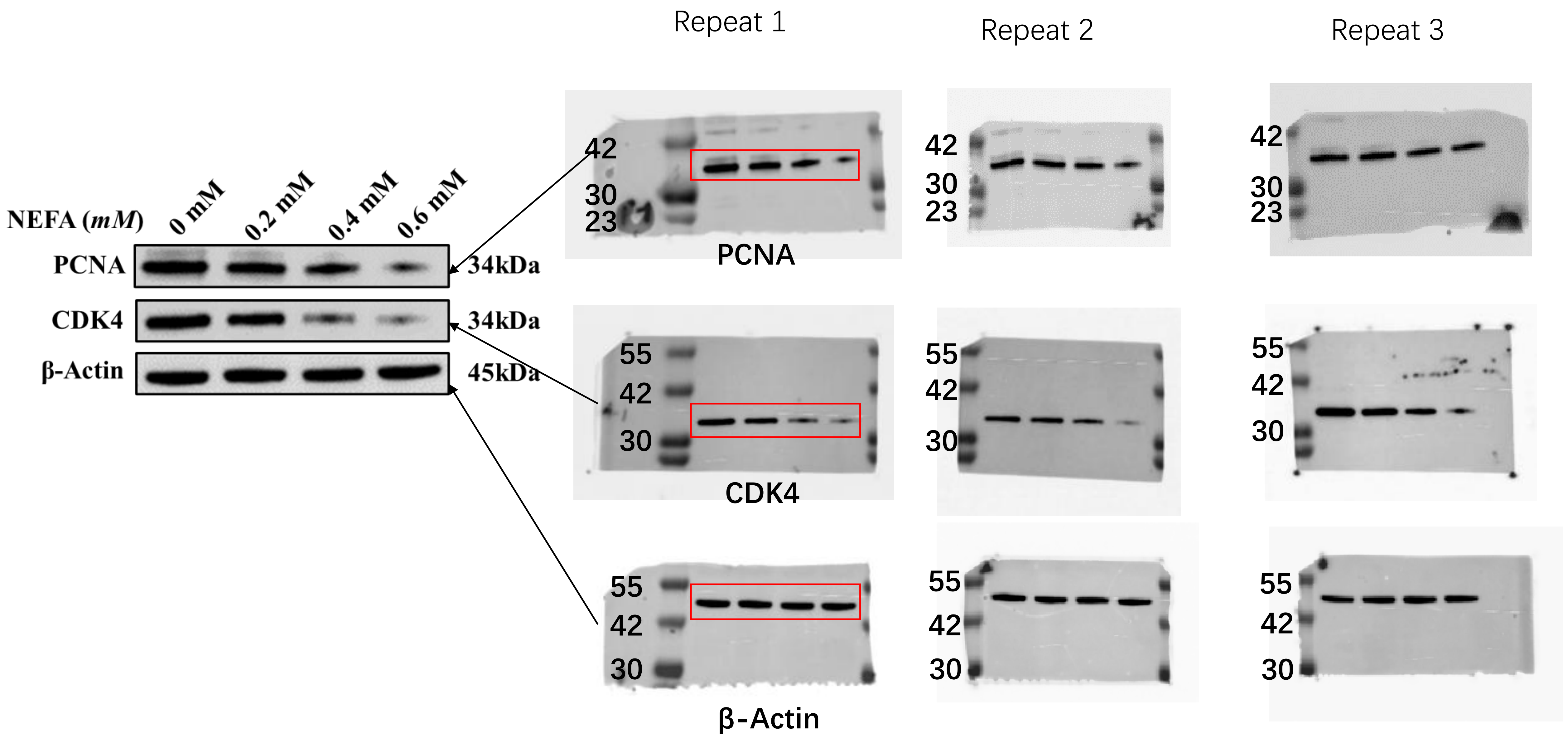

Figure S2. WB original image of Figure 3F

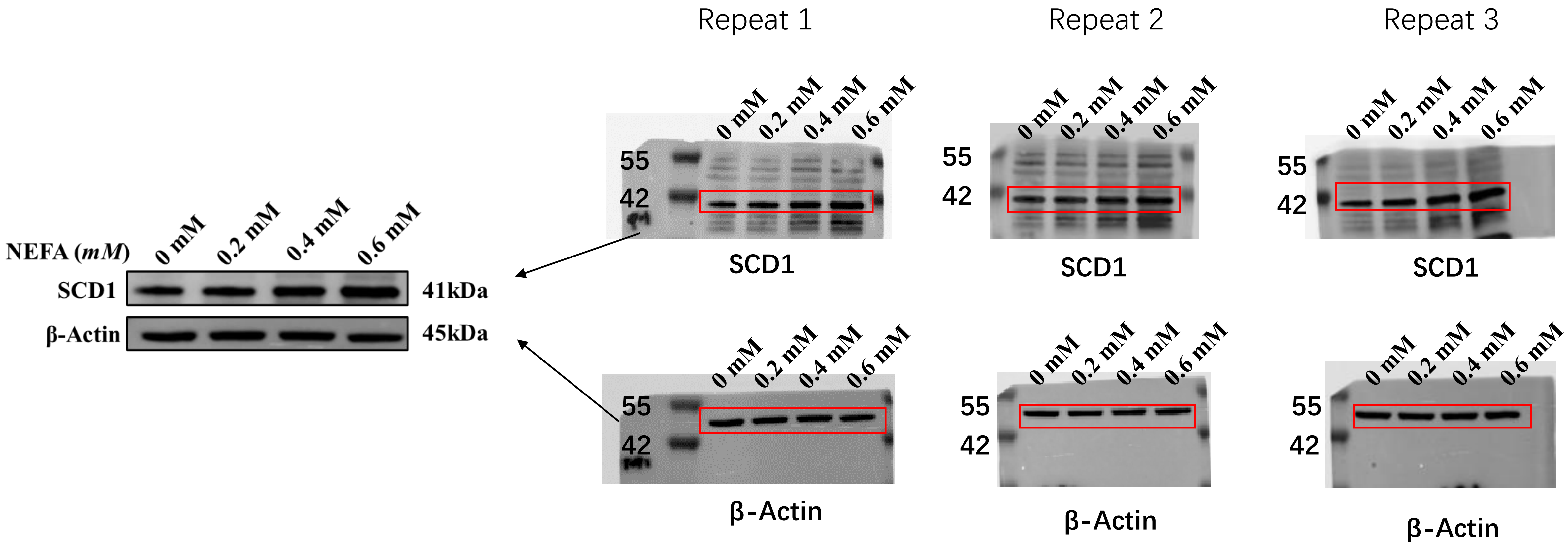

Figure S3. WB original image of Figure 4K

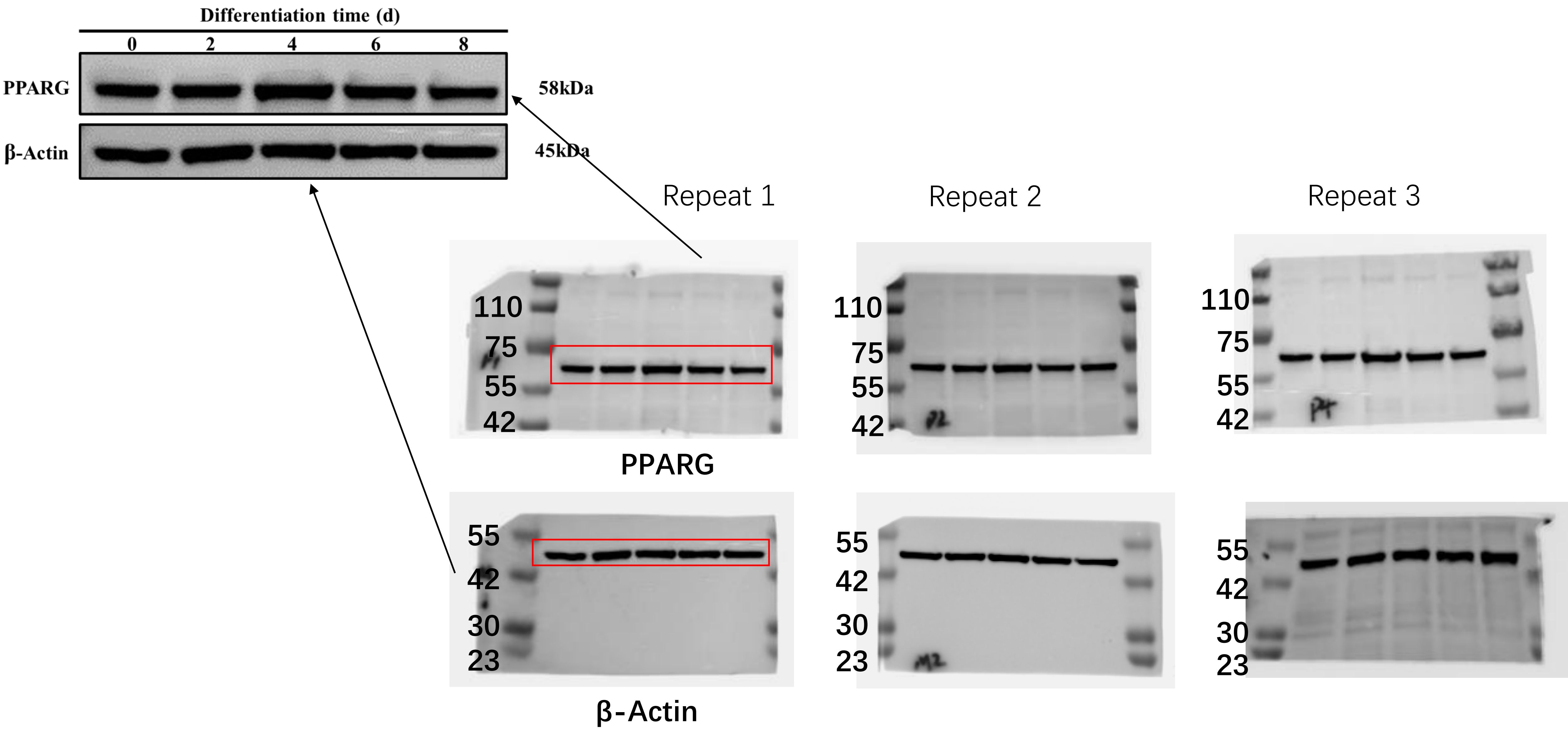

Figure S4. WB original image of Figure 4L

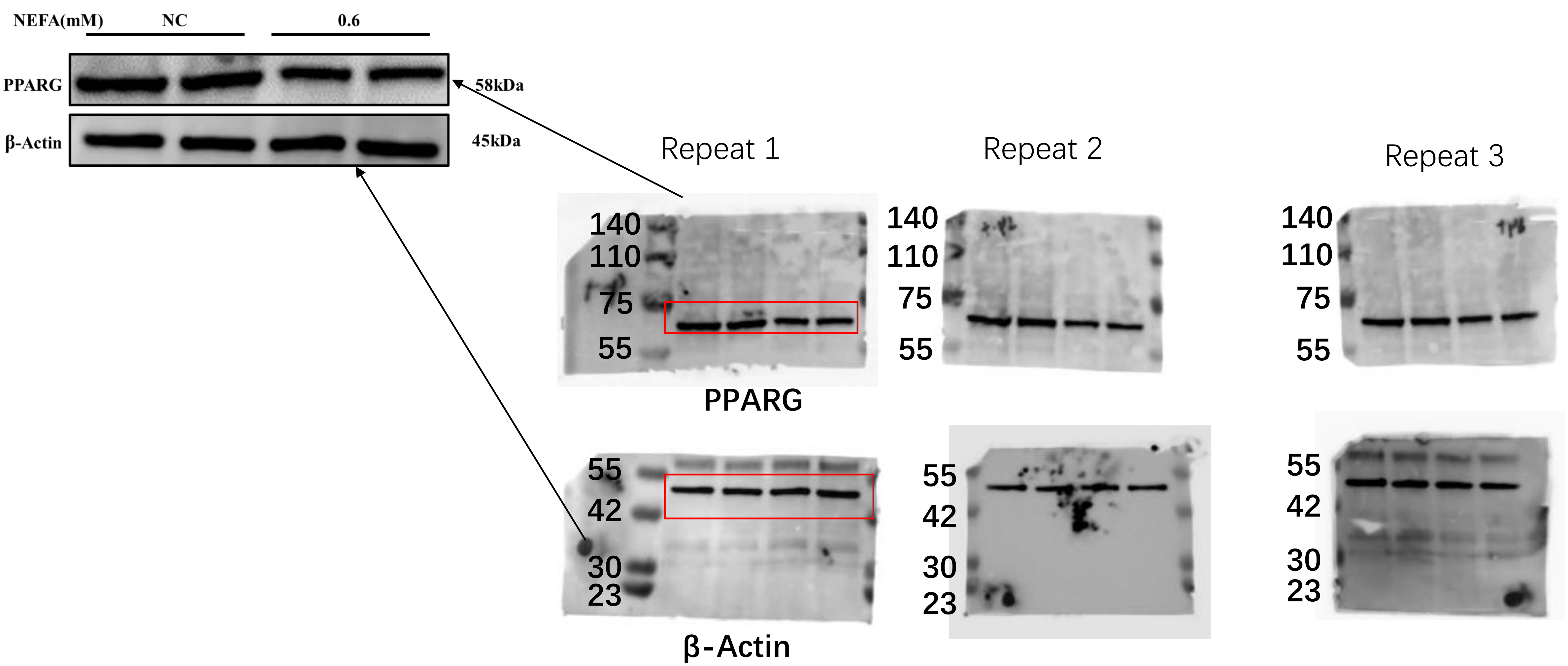

Figure S5. WB original image of Figure 6I

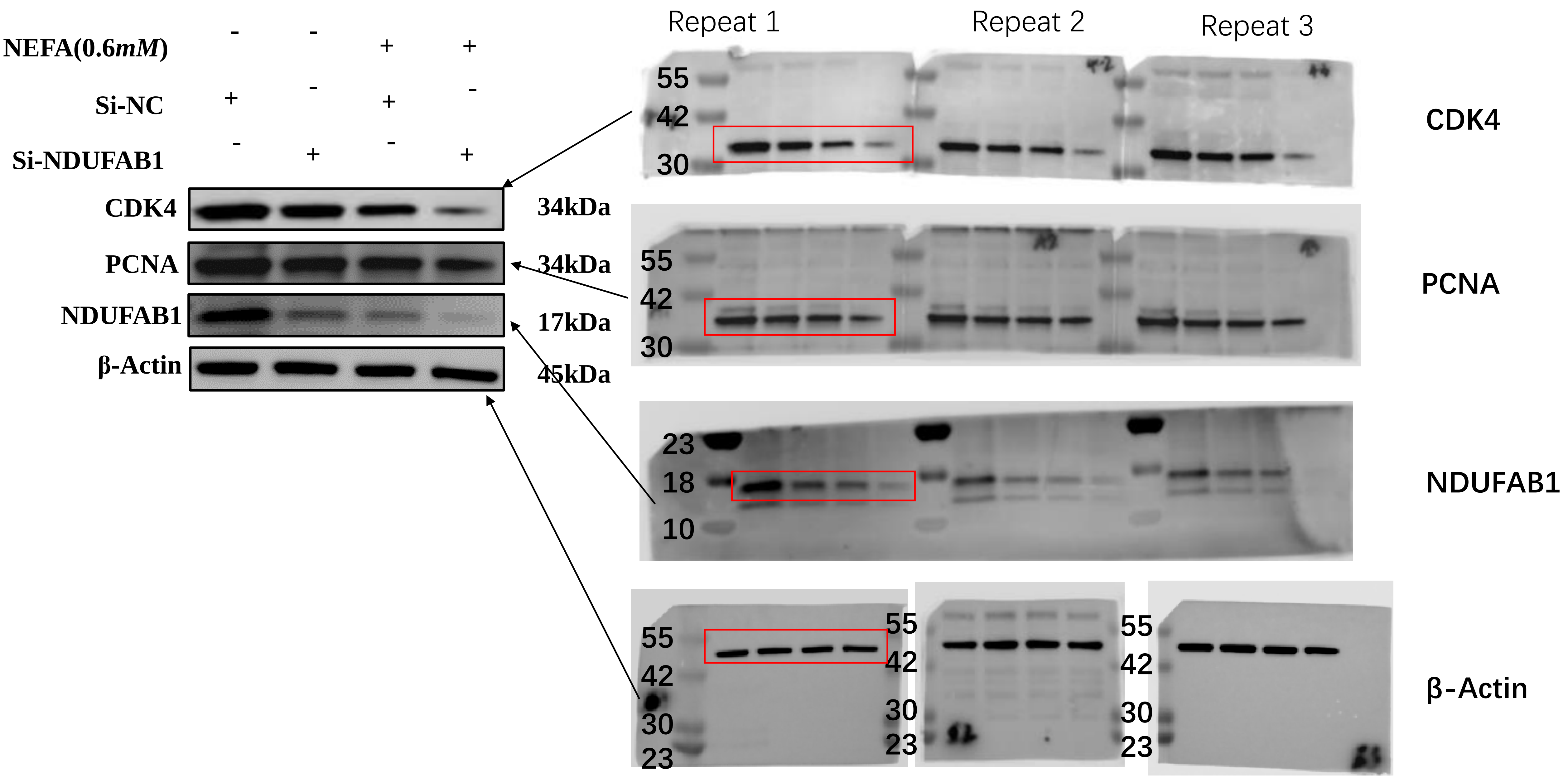

Figure S6. WB original image of Figure 7G

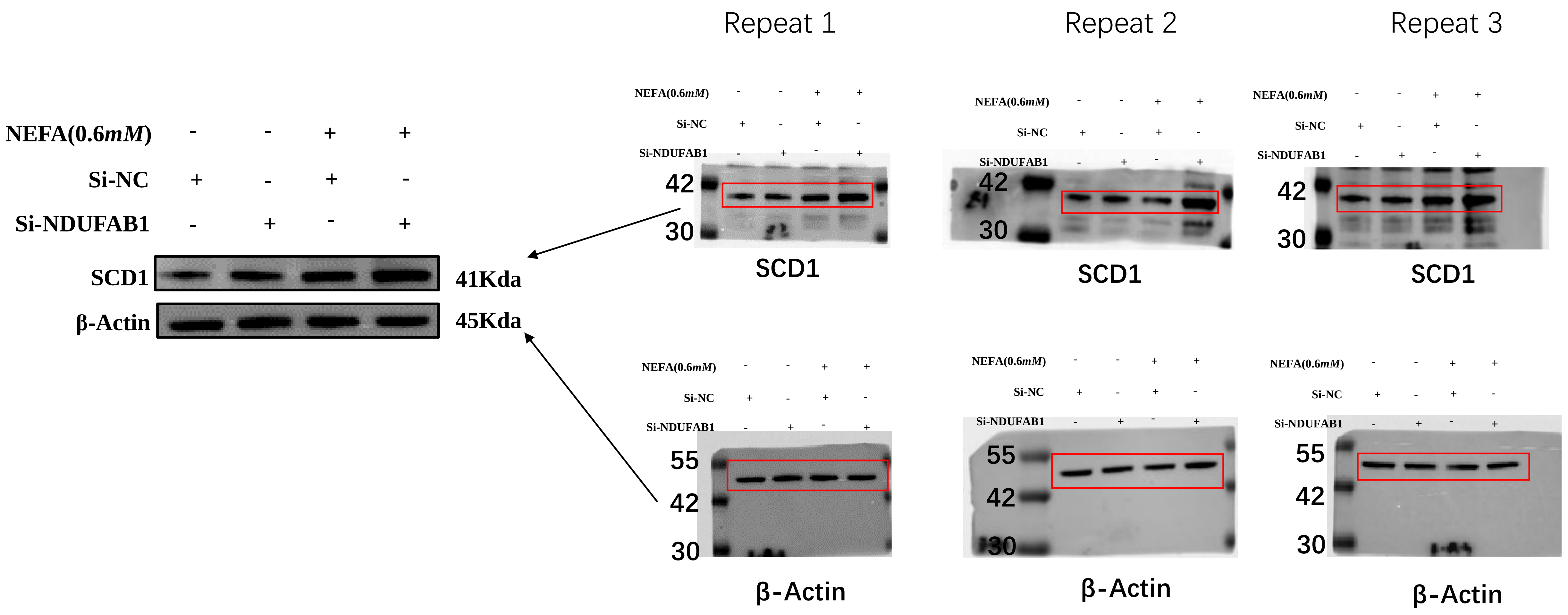

Figure S7. WB original image of Figure 8G

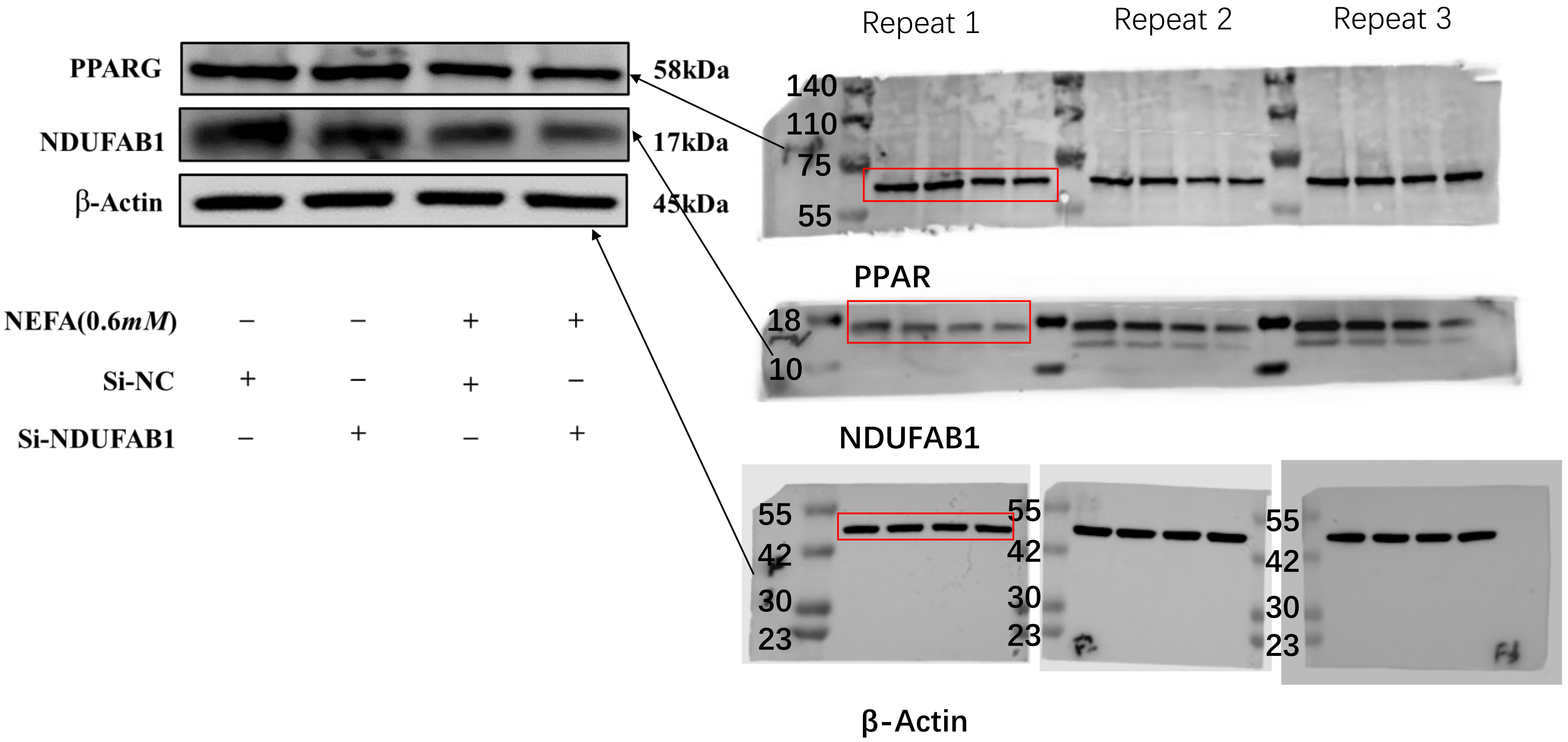

Supplement: Supplementary file 1 [file animals-15-01618-s001.zip › File S1. Original western blot images.pdf]
